# Supplementary material for: Human visual motion perception shows hallmarks of Bayesian structural inference
Source: Sci Rep. 2021 Feb 12;11:3714. doi: 10.1038/s41598-021-82175-7 (PMC7881251; doi:10.1038/s41598-021-82175-7)
Supplement: Supplementary file 1 — Supplementary Information 1. [file 41598_2021_82175_MOESM1_ESM.pdf]

Supplemental figures

Human visual motion perception shows hallmarks of Bayesian structural inference

Sichao Yang, Johannes Bill, Jan Drugowitsch, Samuel J. Gershman

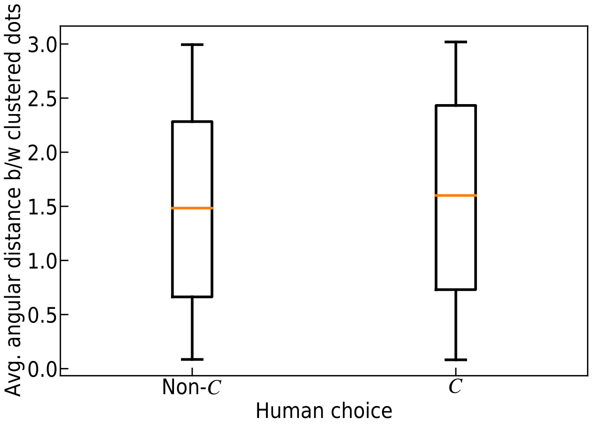

Supplemental Figure S1. Spatial arrangement of dots could influence motion structural inference. We evaluated, for all trials with clustered (C) motion, the average angular distance between the two clustered dots, conditioned on whether participants reported clustered motion or not. The similarity of dot proximity distributions indicates that participants did not rely on dot proximity when solving the task.

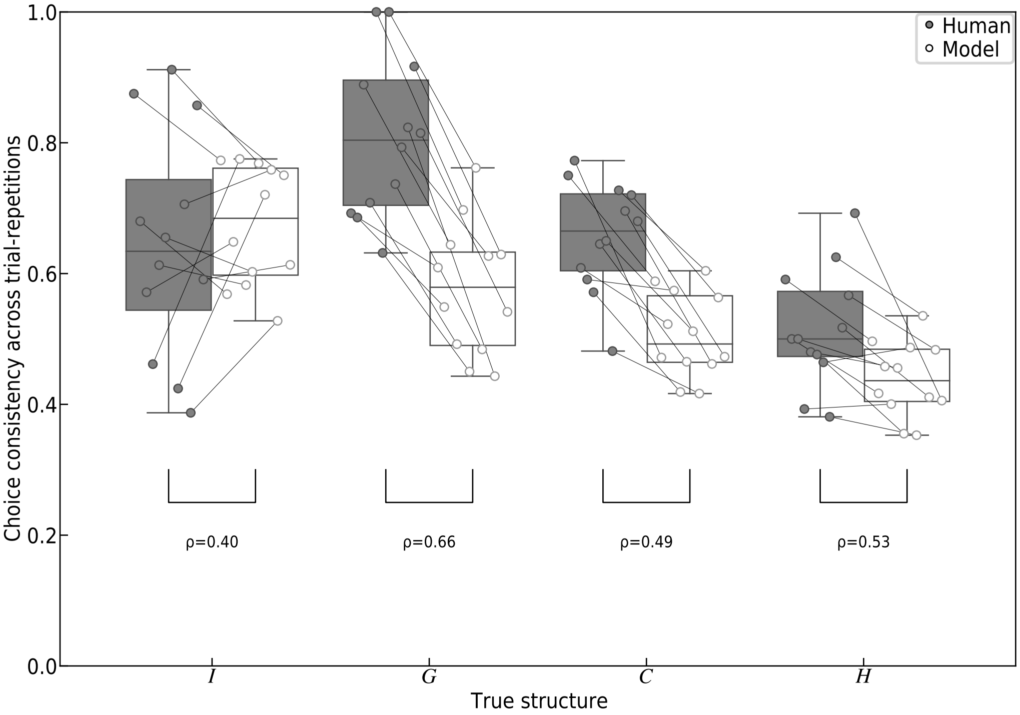

Supplemental Figure S2. Average consistency of human choices and respective model predictions across repetitions of trials, for each of the four structures. Within each structure, the lines link the human and model consistency of each participant and reveal their correlation. This is quantified by the Spearman's rank correlation coefficients ρ in the figure. The model had a tendency to underestimate human consistency, but qualitatively predicted the pattern across conditions. Box plots mark the 0, 25, 50, 75, and 100 percentiles.

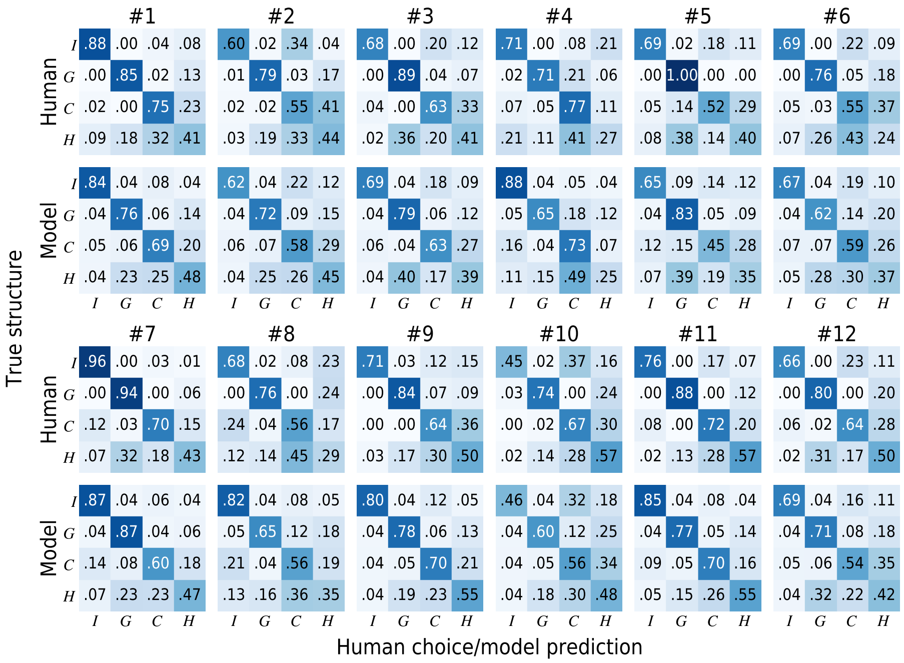

Supplemental Figure S3. Confusion matrices of all twelve participants and their respective model predictions. Participants featured diverse error patters. For example, some participants confused *H*-*G* more than *H*-*C*, while others showed the opposite. The choice model captured the full variety of error patterns.

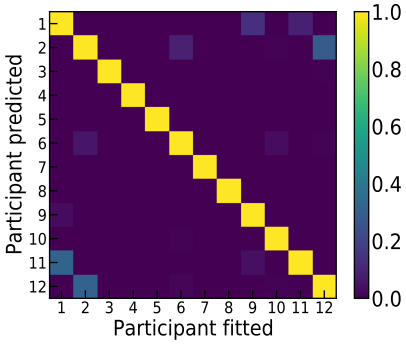

Supplemental Figure S4. Cross-participant model comparison. The (i, j) element is the likelihood of responses of participant i under the parameters fitted to participant j, normalized to 1 within each row. The dominance of diagonal elements implies that individual differences in the confusion matrices were not driven by the set of presented trials.

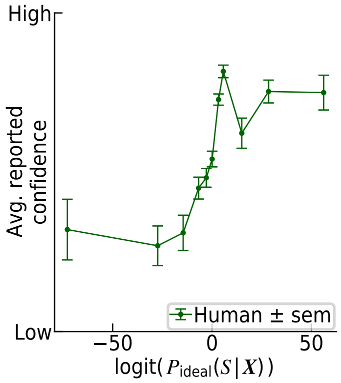

Supplemental Figure S5. Fraction of human high confidence reports as a function of the ideal observer's log-odds, $\text{logit}(P_{\text{ideal}}(S|X))$. Without including the fitted prior (biases b_S), the curve still exhibits an approximately monotonic increase, similar to **Figure 2I**.

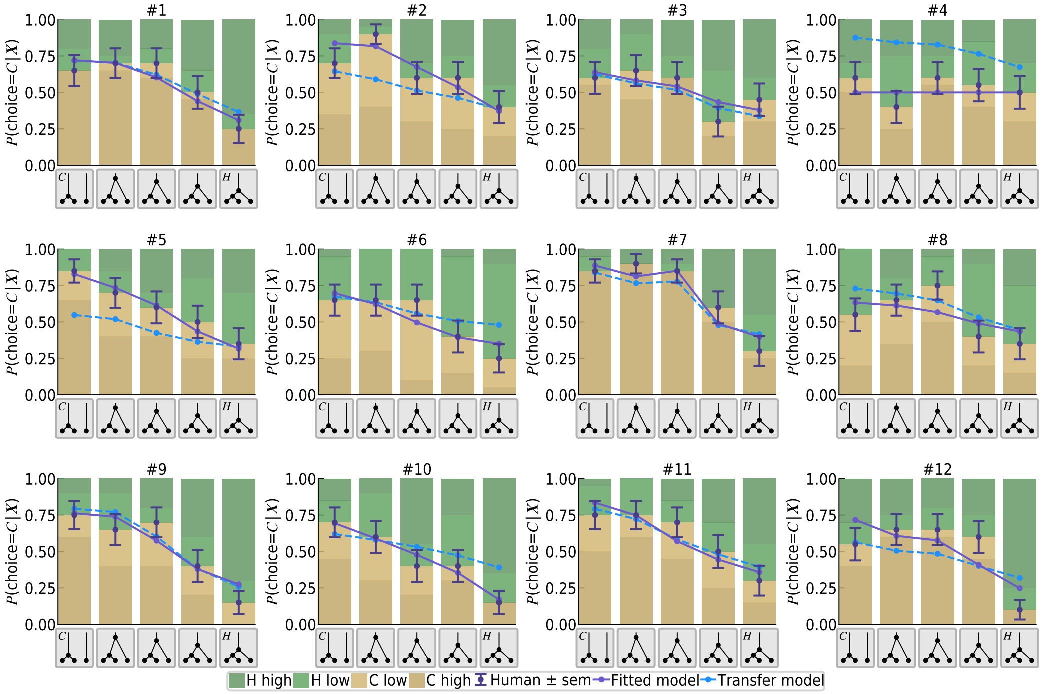

Supplemental Figure S6. Same as **Figure 3B**, but for every participant separately. The fitted model (solid purple line) captured the diverse human transitions of choice, as the structure morphed from C to H. Also, the transfer model (dashed blue line) predicted many participants' responses accurately.

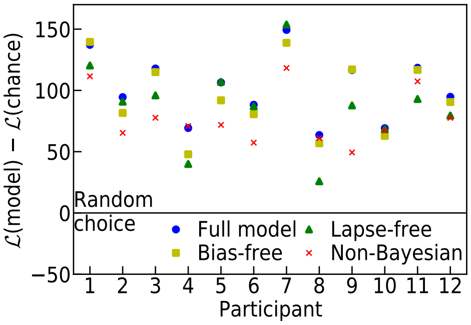

Supplemental Figure S7. Model comparison in terms of the log-likelihood of human responses under the different choice models of **Figure 4A**. All log-likelihoods are evaluated relative to chance level (uniform choice with probability 1/4).

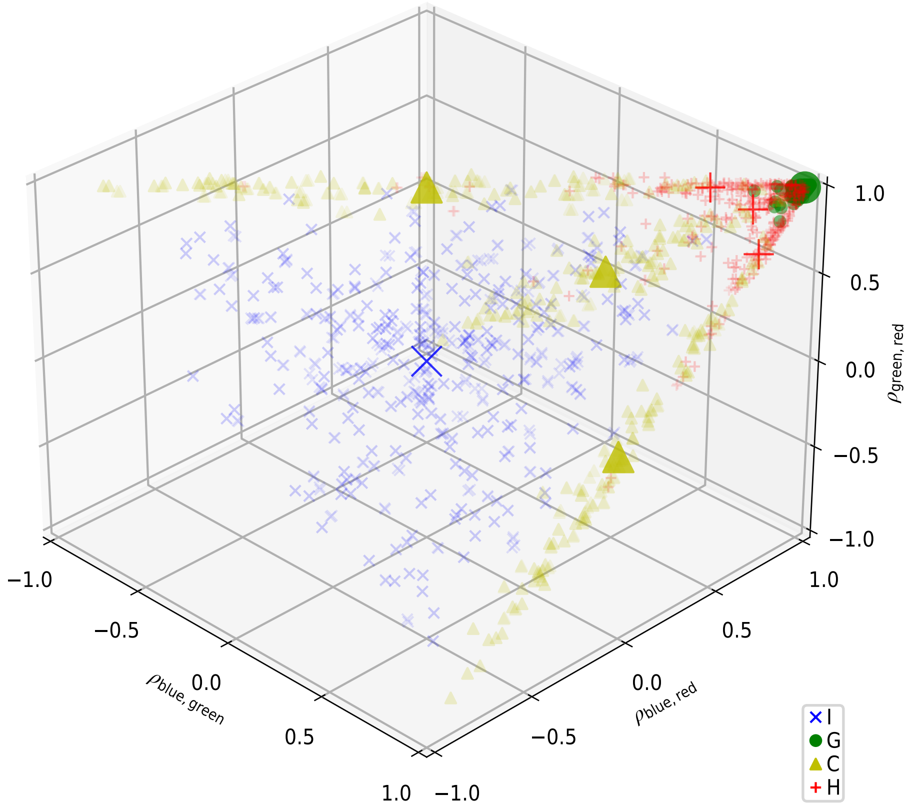

Supplemental Figure S8. Distribution of the three Pearson correlation coefficients $(\rho_{\text{blue,green}}, \rho_{\text{blue,red}}, \rho_{\text{green,red}})$ of the empirical dot velocity trajectories $V(X)$. Colors and markers indicate the generating trial structure. All 1200 unique trials of Experiment 1 are shown. The larger symbols mark the prototype of the motion structures (including multiplicity for C and H). See Materials and Methods for mathematical details about the prototypes and their multiplicity. Trials of the same structure tend to cluster around their prototype, such that decision boundaries could be drawn between different structures, laying the basis for the non-Bayesian, prototype-based model of motion structural inference.
